# The analysis of a reactive hydromagnetic internal heat generating poiseuille fluid flow through a channel

**DOI:** 10.1186/s40064-016-2964-0

**Published:** 2016-08-11

**Authors:** A. R. Hassan, R. Maritz

**Affiliations:** 1 Department of Mathematical Sciences, University of South Africa, Pretoria, 0003 South Africa; 2 Department of Mathematics, Tai Solarin University of Education, Ijagun, Ogun State Nigeria

**Keywords:** Chemical kinetics, Entropy generation, Thermal criticality, Adomian decomposition method (ADM), Pade approximation technique

## Abstract

In this paper, the analysis of a reactive hydromagnetic Poiseuille fluid flow under different chemical kinetics through a channel in the presence of a heat source is carried out. An exothermic reaction is assumed while the concentration of the material is neglected. The Adomian decomposition method together with Pade approximation technique are used to obtain the solutions of the governing nonlinear non-dimensional differential equations. Effects of various physical parameters on the velocity and temperature fields of the fluid flow are investigated. The entropy generation analysis, irreversibility distribution ratio, Bejan number and the conditions for thermal criticality for different chemical kinetics are also presented.

## Background

Considerable effort has been devoted to the study of a reactive hydromagnetic fluid flow which finds numerous and wide-ranging applications in many engineering processes, such as polymer extrusion, nuclear reactor design, geophysics and underground storage of nuclear waste and energy storage systems amongst others. Reactive hydromagnetic fluid flows are often accompanied with heat transfer in many industrial and engineering applications. For instance, Makinde and Beg (2010) devoted their study to investigate the inherent irreversibility and thermal stability in a reactive electrically conducting fluid flowing steadily through a channel with isothermal walls under the influence of a transversely imposed magnetic field. Recently, Hassan and Gbadeyan (2015a) investigated the entropy generation analysis of a reactive hydromagnetic fluid flow through a channel with isothermal wall temperature under different chemical kinetics without taking into account the effects of internal heat generation within the flow system.

A comprehensive survey of the literature (Hassan and Gbadeyan 2013; El-Amin 2004; Patil and Kulkarni 2008; Cortell 2005; Hassan and Gbadeyan 2015b; Saravavan and Kandaswamy 2004; Seddek 2005; Jawdat and Hashim 2010; Oztop and Bilgen 2006; Bagai and Nishad 2012; Di Marcello et al. 2010, Chen 2010; Bartella and Nield 2012) observed that the effects of internal heat generation on a reactive hydromagnetic fluid flow have been studied with respect to various physical properties. For example, Hassan and Gbadeyan (2013) investigated the effect of heat generation on a variable reactive hydromagnetic Couette flow under Arrhenius kinetics and El-Amin (2004) studied the problem of free convection with mass transfer flow in a micro polar fluid bounded by a vertical infinite surface with an exponentially decaying heat generation under the action of a transverse magnetic field. In addition to that the effects of a chemical reaction of first order on a free convective flow of a polar fluid through a porous medium in the presence of internal heat generation was investigated by Patil and Kulkarni (2008), while the heat transfer in a differentially heated, partitioned and square cavity containing heat generating fluid has been studied numerically by Oztop and Bilgen (2006). Also, Jha and Ajibade (2009) investigated the free convective flow of heat generating/absorbing fluid between vertical parallel porous plates due to periodic heating of the porous plates. This analysis was performed by considering a fully developed flow and steady-periodic regime.

It is well known that the rate of heat transfer is temperature dependent, which increases the interaction of moving fluid and thus influence the internal energy of the flow regime. This interaction according to Frank-Kamenettski (1969), Makinde and Beg (2010) as well as Hassan and Gbadeyan (2014) bring about the condition of thermal runaway or ignition in the flow system to predict critical and unsafe situations. In addition to that, Hassan and Gbadeyan (2014) only investigated the thermal criticality of a reactive hydromagnetic fluid flow under different chemical kinetics. Meanwhile, in their study, Hassan and Gbadeyan (2014) together with Makinde and Beg (2010), Hassan and Gbadeyan (2015a) did not consider the effect of the internal heat generation within the flow system, but stated the importance of hydromagnetic reactive flows that are often accompanied with heat transfer, which according to them is an integral part of natural convection flow that belongs to the class of problems in boundary layer theory which occurs in various physical phenomena such as fire engineering, combustion modelling, nuclear reactor, heat exchangers, etc.

Hence, the present study aims to investigate the analysis of a reactive hydromagnetic Poiseuille fluid flow through a horizontal channel under the influence of an internal heat generation produced within the flow system. It is assumed that the reaction is exothermic under different chemical kinetics with their respective numerical exponents (*m*). The analytical solutions of the nonlinear dimensionless equations governing the fluid flow are obtained using the Adomian decomposition method (ADM) together with Pade approximation technique. Also, important properties of velocity and temperature fields including entropy generation analysis and thermal criticality conditions of the fluid flow under different chemical kinetics are discussed. More importantly, our results shall be of interest to industries in improving the efficiency and effectiveness of hydromagnetic lubricants used in engineering systems.

In the rest of this paper, the problem is formulated in “Mathematical formulation” section. The governing equations are solved using the ADM in “The Adomian decomposition method (ADM)” section while the entropy generation analysis and thermal criticality conditions were determined in “Entropy generation” and “Thermal stability and the Pade approximation technique” sections respectively. Presentations of analytical results of the problem are shown in tables, and graphs in “Results and discussion” section and “Conclusion” section gives the concluding remarks.

## Mathematical formulation

**Fig. 1:**
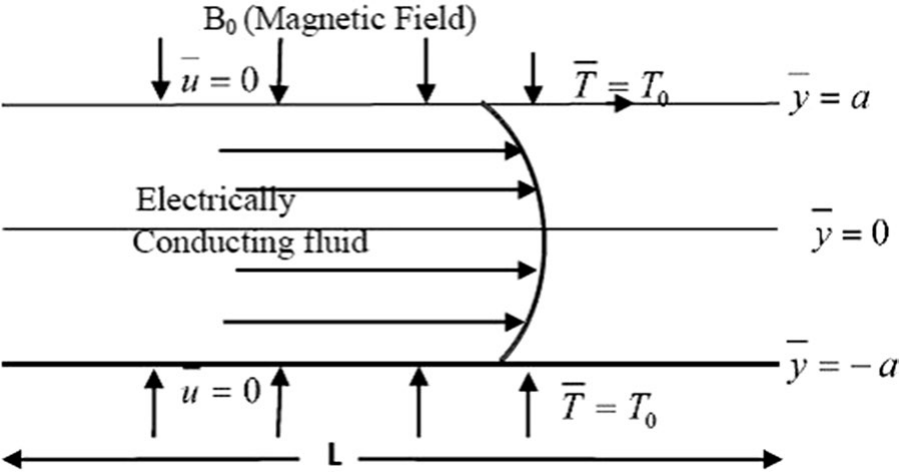
Geometry of the problem

Let us consider the steady flow of an incompressible reactive fluid through a channel made up of two parallel plates with isothermal wall temperature. The fluid is electrically conducted under the influence of a transversely applied magnetic field ($B_0$). The geometry of the problem is shown in Fig. 1 where $\mathbf{L}$ is the channel characteristic length.

The *x*-axis is taken along the centreline of the channel and *y*-axis is transverse to this. The walls of the channel are at distance 2*a* apart. Neglecting the induced magnetic field and the consumption of the reactant, the differential equations governing the fluid flow in non-dimensionless form as in Makinde and Beg (2010), Hassan and Gbadeyan (2015b), Frank-Kamenettski (1969), Jha and Ajibade (2009) may be written as:

1 $$-\frac{\mathrm {d}\overline{p}}{\mathrm {d}\overline{x}}+\mu \frac{\mathrm {d}^2\overline{u}}{\mathrm {d}\overline{y}^2}-\sigma _0 B_0^2\overline{u}=0$$

2 $$\begin{aligned}&k\frac{\mathrm {d}^2\overline{T}}{\mathrm {d}\overline{y}^2}+\mu \left( \frac{\mathrm {d}\overline{u}}{\mathrm {d}\overline{y}}\right) ^2+\sigma _0 B_0^2\overline{u}^2+ QC_0A\left( \frac{k \overline{T}}{\nu \ell }\right) ^m \mathrm {e}^{-{\frac{E}{R\overline{T}}}}+Q_0\left( \overline{T}-T_0\right) =0 \end{aligned}$$

The flow is symmetric about the vertical *x*-axis. Hence the corresponding boundary conditions along the channel centreline is given as:

3 $$\frac{\mathrm {d}\overline{u}}{\mathrm {d}\overline{y}}=\frac{\mathrm {d}\overline{T}}{\mathrm {d}\overline{y}}\quad \text{ on }\quad \overline{y}=0\quad \text{ and }\quad \overline{u}=\overline{T}=0\quad \text{ on }\quad \overline{y}=\pm a.$$

where $\overline{p}$ is the dimensional modified pressure, $\mu$ is fluid viscosity, $\overline{u}$ is the dimensional axial velocity and $\sigma _0$ represents electrical conductivity. Also, *k* represents the thermal conductivity coefficient, $\overline{T}$ is the dimensional fluid temperature, *Q* is the heat of the reaction term, $C_0$ is the reactant species initial concentration, *A* is the reaction rate constant, $\nu$ represents vibration frequency, $\ell$ is Planck’s number and *E* is the activation energy, Also, *R* is the universal gas constant, $Q_0$ is the dimensional heat generation coefficient and $T_0$ is the wall temperature. The numerical exponents $m\in \lbrace -2,0,0.5\rbrace$ respectively represent chemical kinetics for sensitized, Arrhenius and bimolecular kinetics. Finally, it should be noted that the fourth term in (2) is investigated in Makinde and Beg (2010) for only the Arrhenius case (where $m = 0$) and Hassan and Gbadeyan (2014) considered (2) for various kinds of chemical kinetics, but both Makinde and Beg (2010) and Hassan and Gbadeyan (2014) did not consider the last term in (2) which represents the effect of the internal heat generation within the flow system which also is similar to the modelling done in Hassan and Gbadeyan (2015b), Bartella and Nield (2012), Jha and Ajibade (2009).

Introducing the following non-dimensional quantities:

4 $$\begin{aligned}&y=\frac{\overline{y}}{a},\quad x=\frac{\overline{x}}{a},\quad u=\frac{\overline{u}}{U},\quad T=\frac{E\left( \overline{T}-T_0\right) }{R T_0^2},\quad Br=\frac{E \mu U^2}{k R T_0^2}, \quad \delta = \frac{R T_0}{E}, \nonumber \\&\gamma =\frac{\mu U^2}{QAa^2C_0}\left( \frac{\nu \ell }{k T_0}\right) ^m\mathrm {e}^{\frac{E}{RT_0}}, \quad H^2=\frac{\sigma _0 B_0^2 a^2}{\mu }, \quad G=-\frac{\mathrm {d}p}{\mathrm {d}x}, \quad p=\frac{a \overline{p}}{\mu U}, \nonumber \\&\lambda =\frac{QEAa^2C_0}{KRT_0^2}\left( \frac{kT_0}{\nu \ell }\right) ^m\mathrm {e}^{-\frac{E}{RT_0}}\quad \text{ and }\quad \beta =\frac{Q_0RT_0^2}{QAEC_0}\left( \frac{\nu \ell }{k T_0}\right) ^m\mathrm {e}^{\frac{E}{RT_0}}, \end{aligned}$$

the governing boundary value problem equations (1)–(3) in dimensionless form become:

5 $$G+\frac{\mathrm {d^2}u}{\mathrm {d}y^2}-H^2u=0$$

6 $$\frac{\mathrm {d^2}T}{\mathrm {d}y^2}+\lambda \left[ \left( 1+\delta T\right) ^m \mathrm {e}^{\frac{T}{1+\delta T}}+\gamma \left( \left( \frac{\mathrm {d}u}{\mathrm {d}y}\right) ^2+H^2u^2\right) +\beta T\right] =0$$

together with the boundary conditions

7 $$\frac{\mathrm {d}u}{\mathrm {d}y}=\frac{\mathrm {d}T}{\mathrm {d}y}\quad \text{ on }\quad y=0\quad \text{ and }\quad u = T =0 \quad \text{ on }\quad y=\pm 1.$$

where the non-dimensional variables *u* is the axial velocity and *T* is the fluid temperature. Also, other parameters include *G*, which represent the pressure gradient, *a* is the channel half width, *U* is the mean velocity, *H* is the Hartmann number, $\lambda$ is the critical explosion parameter named after Frank-Kamenettski, *Br* is the Brinkman number, $\delta$ is the activation energy parameter, $\gamma$ represents the viscous heating parameter and $\beta$ is the heat source parameter.

## The Adomian decomposition method (ADM)

As already mentioned, the non-dimensional non-linear coupled boundary value problems (5)–(7) governing the flow of a reactive magnetohydrodynamics internal heat generating Poiseuille fluid is solved in this section using the ADM (Hassan and Gbadeyan 2013, 2014; Wazwaz and El-Sayed 2001; Hassan and Fenuga 2011; Adesanya and Gbadeyan 2011; Gbadeyan and Hassan 2012; Kutafina 2011).

In order to decouple the boundary value problems, Eq. (5) is a linear second order non-homogeneous differential equation that can be solved by splitting it into a complimentary function and a particular integral; together with appropriate boundary conditions to give a general exact solution as:

8 $$u(y)=\frac{G}{H^2}\left( 1-\frac{\cosh [H y]}{\cosh [H]}\right) .$$

Substituting (8) into the energy equation (6), one obtains the following uncoupled boundary value problem composed of a second order differential equation

9 $$\frac{\mathrm {d^2}T}{\mathrm {d}y^2}+\lambda \left[ \left( 1+\delta T\right) ^m\mathrm {e}^{\frac{T}{1+\delta T}}+\frac{\gamma G^2}{H^2}\left( \frac{\cosh [2 H y]}{\cosh ^2 H}-\frac{2 \cosh [H y]}{\cosh [H]}+1\right) +\beta T\right] =0$$

and the boundary conditions

10 $$\frac{\mathrm {d}T}{\mathrm {d}y}\left( 0\right) =0\quad \text{ and } \quad T(1)=0$$

We now solve the boundary value problem for various types of chemical kinetics as follows:

11 $$L(T)=\frac{\mathrm {d^2}T}{\mathrm {d}y^2}+\lambda \left[ \left( 1+\delta T\right) ^m\mathrm {e}^{\frac{T}{1+\delta T}}+\frac{\gamma G^2}{H^2}\left( \frac{\cosh [2 H y]}{\cosh ^2 H}-\frac{2 \cosh [H y]}{\cosh [H]}+1\right) +\beta T\right] =0$$

where *L* is a second order differential operator. Hence

12 $$L^{-1}=\int _0^y\int _0^y(\bullet )\mathrm {d}y\; \mathrm {d}y$$

Applying (12) to both sides of (11), we have

13 $$T(y)=a_0-\lambda \int _0^y\int _0^y\left[ \left( 1+\delta T\right) ^m\mathrm {e}^{\frac{T}{1+\delta T}}+\frac{\gamma G^2}{H^2}\left( \frac{\cosh [2 H y]}{\cosh ^2 H}-\frac{2 \cosh [H y]}{\cosh [H]}+1\right) +\beta T\right] \mathrm {d}y\;\mathrm {d}y$$

where $a_0 = T(0)$ is to be determined by using the other boundary condition. The ADM requires that the approximate solution is the partial sum:

$$T(y)=\sum _{n=0}^k T_n (y)$$

of the following series

14 $$T(y)=\sum _{n=0}^\infty T_n (y)$$

where the components $T_0, T_1, T_2, \ldots ,T_k$ are to be determined. Thus substituting (14) into (13) we have

15 $$\begin{aligned}T(y)&=a_0-\lambda \int _0^y\int _0^y\left[ \left( 1+\delta \sum _{n=0}^\infty T_n (y)\right) ^m\mathrm {e}^{\frac{\sum _{n=0}^\infty T_n (y)}{1+\delta \sum _{n=0}^\infty T_n (y)}}\right. \nonumber \\&\ \quad \left. +\frac{\gamma G^2}{H^2}\left( \frac{\cosh [2 H y]}{\cosh ^2 H}-\frac{2 \cosh [H y]}{\cosh [H]}+1\right) +\beta \sum _{n=0}^\infty T_n (y)\right] \mathrm {d}y\;\mathrm {d}y \end{aligned}$$

To determine the components $T_0, T_1, T_2, \ldots ,T_k$, we let the non-linear term be represented by the following series:

16 $$\sum _{n=0}^\infty A_n(y)=\left( 1+\delta \sum _{n=0}^\infty T_n (y)\right) ^m\mathrm {e}^{\frac{\sum _{n=0}^\infty T_n (y)}{1+\delta \sum _{n=0}^\infty T_n (y)}}$$

whose components $A_0, A_1, A_2, \ldots\,,$ are called Adomian polynomials. Then, (16) is thereby expanded such that

17 $$\begin{aligned}A_0&=\mathrm {e}^{\frac{T_0(y)}{1+\delta T_0 (y)}}\left[ 1+\delta T_0(y)\right] ^m, \\A_1&=\mathrm {e}^{\frac{T_0(y)}{1+\delta T_0 (y)}}\left[ 1+\delta T_0(y)\right] ^{-2+m}\left( 1+m\delta +m\delta ^2 T_0(y)\right) T_1(y), \\A_2&=\frac{1}{2}\mathrm {e}^{\frac{T_0(y)}{1+\delta T_0 (y)}}\left( 1+2(m-1)\delta + (m-1)m \delta ^2 + 2(m-1)\delta ^2(1+m\delta )T_0(y)\right. \nonumber \\&\quad \left. +(m-1)m\delta ^4 T_0(y)^2\right) T_1(y)^2+\left( 2(1+\delta T_0(y)^2(1+m \delta +m \delta ^2 T_0(y)T_2(y)\right) ,\ldots, \end{aligned}$$

Also, (15) reduces to

18 $$T(y)=a_0-\lambda \int _0^y\int _0^y\left[ \left( \sum _{n=0}^\infty A_n(y)\right) +\frac{\gamma G^2}{H^2}\left( \frac{\cosh [2 H y]}{\cosh ^2 H}-\frac{2 \cosh [H y]}{\cosh [H]}+1\right) +\beta \sum _{n=0}^\infty T_n (y)\right] \mathrm {d}y\;\mathrm {d}y$$

Following Wazwaz and El-Sayed (2001), Hassan and Fenuga (2011), Adesanya and Gbadeyan (2011), we take the zeroth component of (18) and we obtain the following

19 $$T_0(y)= a_0$$

20 $$T_1(y)=-\lambda \int _0^y\int _0^y\left[ \left( A_0(y)\right) +\frac{\gamma G^2}{H^2}\left( \frac{\cosh [2 H y]}{\cosh ^2 H}-\frac{2 \cosh [H y]}{\cosh [H]}+1\right) +\beta T_0 (y)\right] \mathrm {d}y\;\mathrm {d}y$$

21 $$T_{n+1}(y)=-\lambda \int _0^y\int _0^y\left[ A_n(y)+\beta T_n\right] \mathrm {d}y\;\mathrm {d}y, \qquad \qquad n\ge 1$$

Hence, the approximate solution of the boundary value problem is obtained as

22 $$T(y)=\sum _{n=0}^k T_n(y)$$

Equations (19)–(21) are then coded in the Mathematica software package to obtain the approximate solutions used and discussed in the next sections.

## Entropy generation

Entropy generation is a measure of the account of irreversibility associated with the real process. It is a measure of disorderliness in a system. In order to preserve the quality of energy in a fluid flow process or at least to reduce the entropy generation, it is also important to study the distribution of the entropy generation within the fluid volume. Hence, in this section, the analysis of entropy generation of a reactive hydromagnetic internal heat generating fluid flow is discussed. According to Hassan and Gbadeyan (2015a) and Wood (1975), the general equation for the entropy generation per unit volume in the presence of the magnetic field is given as:

23 $$S^m=\frac{k}{T_0^2}\left( \frac{\mathrm {d}\overline{T}}{\mathrm {d}\overline{y}}\right) ^2 +\frac{\mu }{T_0}\left( \frac{\mathrm {d}\overline{u}}{\mathrm {d}\overline{y}}\right) ^2+\frac{\sigma _0 B_0^2 \overline{u}^2}{T_0}$$

The first term of $S^m$ in (23) is the irreversibility due to heat transfer, the second term is the entropy generation due to viscous dissipation and the third term is the local entropy generation due to the effect of the magnetic field. The dimensionless form of the entropy generation number ($S^m$ ), using the dimensionless variables and parameters in Eq. (4) is obtained as:

24 $$N_s=\frac{S^m a^2 E^2}{k R^2 T_0^2}=\left( \frac{\mathrm {d} T}{\mathrm {d} y}\right) ^2+\frac{Br}{\Omega }\left[ \left( \frac{\mathrm {d} u}{\mathrm {d} y}\right) ^2+H^2 u^2\right]$$

The first term of $N_s$, $\left( \frac{\mathrm {d} T}{\mathrm {d} y}\right) ^2$ is assigned $N_1$ which represents the irreversibility due to heat transfer while the second term $\frac{Br}{\Omega }\left[ \left( \frac{\mathrm {d} u}{\mathrm {d} y}\right) ^2+H^2 u^2\right]$, referred to as $N_2$ is the entropy generation due to the combined effects of the viscous dissipation and the magnetic field, where $\Omega =RT_0/E$ is the wall temperature parameter.

Now, we let

25 $$\phi =\frac{N_1}{N_2}$$

denote the irreversibility distribution ratio. Relation (25) shows that heat transfer dominates when $0\le \phi < 1$ and fluid friction dominates when $\phi > 1$. This is used to determine the contribution of heat transfer in many engineering designs. As an alternative to the irreversibility parameter, the Bejan number (*Be*) is defined as

26 $$Be=\frac{N_1}{N_s}=\frac{1}{1+\phi } \quad \text{ where } \quad 0\le Be\le 1$$

The results of the computation of the entropy analysis are shown in Table 2 as well as in Figs. 5 and 6. The details of the results are thereby discussed in “Results and discussion” section.

## Thermal stability and the Pade approximation technique

The analysis of the thermal criticality for different chemical kinetics under the influence of a magnetic intensity field and heat source is carried out in this section. Firstly, the expressions for the unknown constant $(a_0)$ are computed. This is done by using the Pade approximation technique to obtain the solutions of the non-linear boundary value problem equations governing the fluid flow. To this end, the diagonal form of the series solution (22) is evaluated respectively at $y = 1$ using the built-in Pade approximant procedure in the mathematica software package with the boundary condition (10).

Taking the diagonal Pade approximant of (22) at various values lead to an eigenvalue problem. To show that the series converges, the unknown constant $(a_0)$ is evaluated using values for the known parameters for any of the chemical kinetics. The critical values of the Frank-Kamenettski parameter $(\lambda _c )$ for the non-existence of the solution, or the thermal runaway for each chemical kinetics are presented and discussed in the next section.

## Results and discussion

In this section, we compare the solutions of temperature profiles, entropy generation rates, solution branches and thermal criticality for different chemical kinetics under the influence of a heat source and magnetic intensity. However, our results shall show the efficiency of the ADM and the effect of internal heat generation which was not accounted for in Makinde and Beg (2010) where the Perturbation method (PM) was used to find the solutions of the governing equations. Notably, our results shall be equivalent to that of Makinde and Beg (2010) when the numerical exponent (*m*) and internal heat generation term $(\beta )$ in our results are both zero.

**Table 1:** Comparison of numerical results of the temperature profile

$$H=1,G=1,\delta =1,\gamma =1,\lambda =0.5$$				
*y*	*PM* (Makinde and Beg 2010)	$ADM (\beta =0)$	Absolute error	$ADM (\beta =0.5)$
−1.0	0	0.0002178316	$2.1783 \times 10^{-4}$	0.0003681026
−0.75	0.1556934861	0.1577379743	$2.0445\times 10^{-3}$	0.1771365903
−0.50	0.2660663845	0.2691056991	$3.0396\times 10^{-3}$	0.3046016196
−0.25	0.3323243479	0.3358417798	$3.5177\times 10^{-3}$	0.3819272135
0	0.3544502181	0.3581076494	$3.6578\times 10^{-3}$	0.4078774354
0.25	0.3323243479	0.3358417798	$3.5177\times 10^{-3}$	0.3819272135
0.50	0.2660663845	0.2691056991	$3.0396\times 10^{-3}$	0.3046016196
0.75	0.1556934861	0.1577379743	$2.0445\times 10^{-3}$	0.1771365903
1.0	0	0.0002178316	$2.1783 \times 10^{-4}$	0.0003681026

**Table 2:** Computation of the entropy analysis for different kinetics

$$H=1,G=1,Br=10,\alpha =0.1,\Omega =0.1$$										
	$N_1$			$N_2$	$\phi =\frac{N_1}{N_2}$			$Be=\frac{1}{1+\phi }$		
*y*	$m=-2$	$m=0$	$m=0.5$	$N_2$	$m=-2$	$m=0$	$m=0.5$	$m=-2$	$m=0$	$m=0.5$
$-1$	0.3366	0.6505	0.8605	0.580026	1.7234	0.8917	0.6741	0.3672	0.5286	0.5974
$-0.75$	0.1664	0.3703	0.5282	0.309902	1.8619	0.8368	0.5901	0.3494	0.5444	0.6289
$-0.5$	0.0688	0.1685	0.2499	0.186529	2.7105	1.1070	0.7465	0.2695	0.4746	0.5726
$-0.25$	0.0166	0.0429	0.0653	0.136751	8.2356	3.1844	2.0949	0.1083	0.2390	0.3231
0	0	0	0	0.123866	$\infty$	$\infty$	$\infty$	0	0	0
0.25	0.0166	0.0429	0.0653	0.136751	8.2356	3.1844	2.0949	0.1083	0.2390	0.3231
0.5	0.0688	0.1685	0.2499	0.186529	2.7105	1.1070	0.7465	0.2695	0.4746	0.5726
0.75	0.1664	0.3703	0.5282	0.309902	1.8619	0.8368	0.5901	0.3494	0.5444	0.6289
1	0.3366	0.6505	0.8605	0.580026	1.7234	0.8917	0.6741	0.3672	0.5286	0.5974

Table 1 shows the comparison of numerical results of the temperature profile between the ADM and the PM used in Makinde and Beg (2010). The results showed the efficiency of the ADM as another alternative in getting approximate solutions to differential equations with average differences of order $10^{-3}$. Also, the results showed the effect of the internal heat generation parameter $(\beta )$ as it increases from 0 to 0.5; that is, an increase is noticed in the fluid temperature at both ends of the wall and the maximum temperature is noticed at the centreline of the fluid channel.

Table 2 shows the computation of the entropy generation analysis for different chemical kinetics and shows that fluid friction dominates at the core region of the flow at the upper and lower surfaces of the plate as $\phi > 1$. Also Bejan numbers (*Be*) lie between 0 and 1 for the three chemical kinetics. The effects of the heat source and magnetic intensity are significantly compared to results where there are no heat source and magnetic intensity respectively.

**Table 3:** Numerical values of $a_0$ for sensitized kinetics $(m=-2)$

*Pade*	*H*	$\lambda$	$\beta$	$T_{lower}$	$T_{upper}$
2 / 2	1	0.5	0.5	$-0.3330$	0.271173
5 / 5	1	0.5	0.5	$-0.7249$	0.270982
10 / 10	1	0.5	0.5	$-1.8892$	0.270982
15 / 15	1	0.5	0.5	$-1.8715$	0.270982
20 / 20	1	0.5	0.5	$-1.8715$	0.270982
25 / 25	1	0.5	0.5	$-1.8715$	0.270982
30 / 30	1	0.5	0.5	$-1.8715$	0.270982
50 / 50	1	0.5	0.5	$-1.8715$	0.270982

Table 3 represents the rapid convergence of the ADM for obtaining the minimum and maximum temperature for sensitized chemical kinetics when ($m=-2$) which represents the upper and lower solutions of the flow system.

Table 4 shows the effect of viscous heating and magnetic intensity on the development of thermal runaway for different chemical kinetics. An increase in the viscous heating $(\gamma )$ gives an increase in the critical values $(\lambda _c)$ of Frank-Kamenettski parameters in sensitized and bimolecular kinetics whereas under Arrhenius kinetics, a reduction is noticed. Also, an increase in magnetic intensity (*H*) gives an increase in the critical values $(\lambda _c)$ of Frank-Kamenettski parameters in Arrhenius and bimolecular kinetics while a reduction is observed in sensitized kinetics.

**Table 4:** Effects of different parameters on the development of thermal runaway

*Pade*	*H*	$\gamma$	$\delta$	*G*	$\beta$	$\lambda _c(m=-2)$	$\lambda _c(m=0)$	$\lambda _c(m=0.5)$
2/2	1	1	1	1	0.5	−1.7811263742438508	1.0523144807452944	1.2366868184966630
2/2	1	2	1	1	0.5	−1.6241610492525835	0.9575639392658895	1.2326845092486176
2/2	1	3	1	1	0.5	−1.4923147215659487	0.8785368728933426	1.2284706885201377
2/2	1	1	1	1	0.5	−1.7811263742438508	1.0523144807452944	1.2366868184966630
2/2	2	1	1	1	0.5	−1.8283359751229398	1.0788970932308604	1.2370083898323496
2/2	3	1	1	1	0.5	−1.8785238395429784	1.1097801101901550	1.2381827113700314

 The effect of the internal heat generation on the critical values $(\lambda _c)$ of Frank-Kamenettski parameters compared with previously obtained results in Hassan and Gbadeyan (2014) where the impact of internal heat generation was not considered, that is, when $\beta =0$ is displayed in Table 5. The new results where ($\beta = 0.5$) showed that the thermal critical values $(\lambda _c)$ reduce for each chemical kinetics over the influence of internal heat generation parameter ($\beta$) from 0 to 0.5. Whereas, in both cases, the critical values $(\lambda _c)$ of Frank-Kamenettski parameters increases as the numerical exponent *m* increases from $m = -2$ to $m = 0.5$.

**Table 5:** Effects of internal heat generation on thermal stability compared with Hassan and Gbadeyan (2014)

*Pade*	*m*	*H*	$\gamma$	$\delta$	*G*	$\lambda _c$ (Hassan and Gbadeyan 2014) $(\beta =0)$	$\lambda _c (\beta =0.5)$
2/2	−2	1	1	1	1	−0.2037	−1.7811
2/2	0	1	1	1	1	1.2960	1.0523
2/2	0.5	1	1	1	1	1.6170	1.2367

**Fig. 2:**
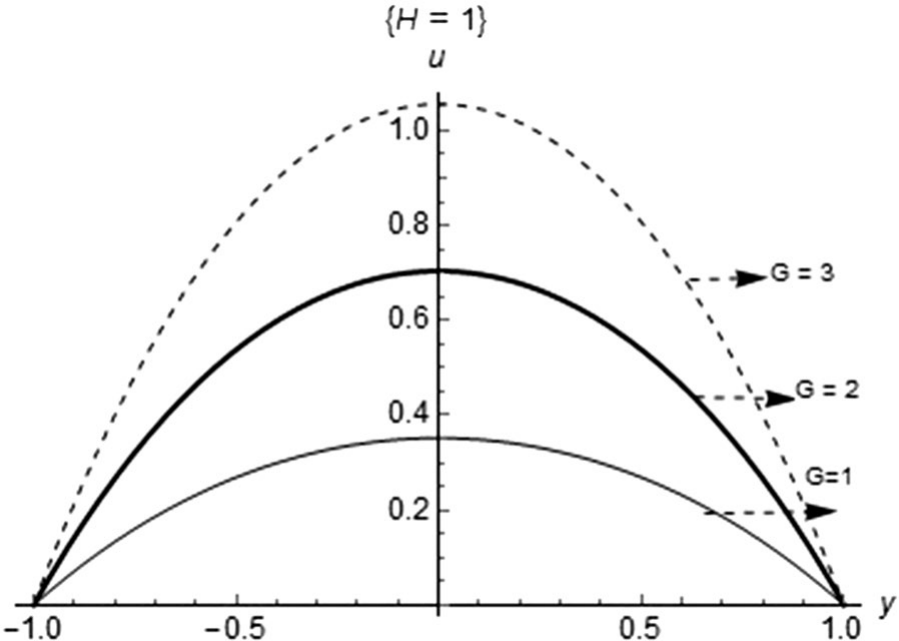
Fluid velocity profile with variations in the pressure gradient

**Fig. 3:**
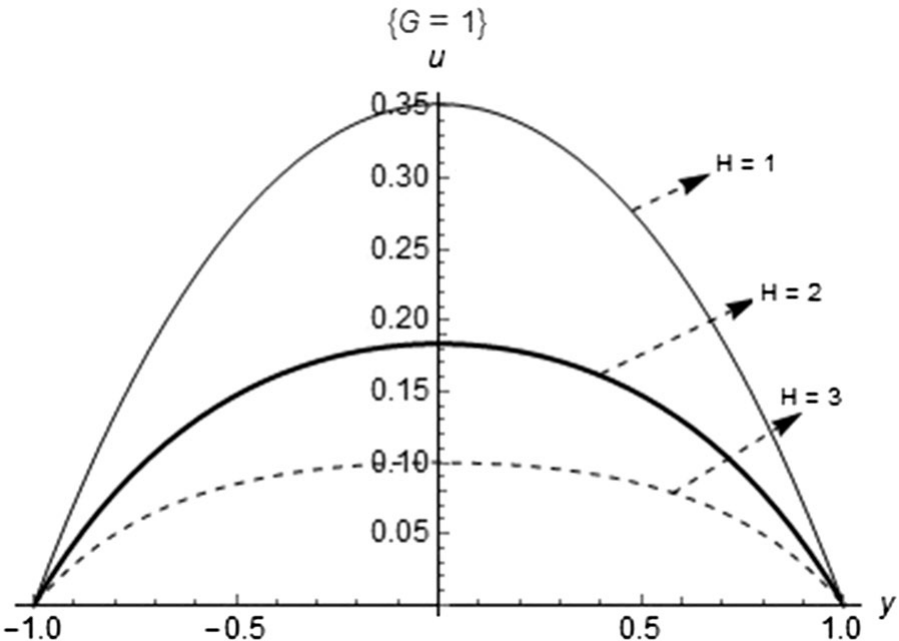
Fluid velocity profile with variations in magnetic field intensity

Figure 2 shows the effect of pressure gradient (*G*) on the fluid velocity. The maximum velocity occurs as the pressure gradient (*G*) increases. This is true in the sense that, the more the pressure is applied in the channel, the faster the flow of the fluid. The plot of the velocity profile for variations in the Hartmann number (*H*) is shown in Fig. 3. As observed, the maximum velocity occurs at the minimum value of the parameter. Further increase in (*H*) decreases the flow velocity maximum; this is due to the presence of Lorentz forces which has retarding effects on fluid flow when placed across the flow channel.

**Fig. 4:**
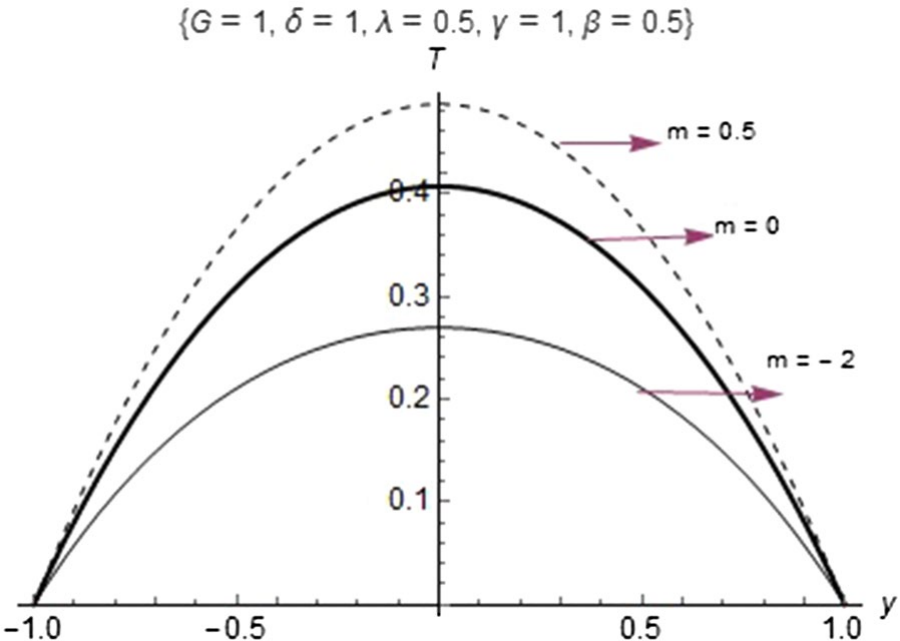
Comparison of fluid temperature profiles for different kinetics

**Fig. 5:**
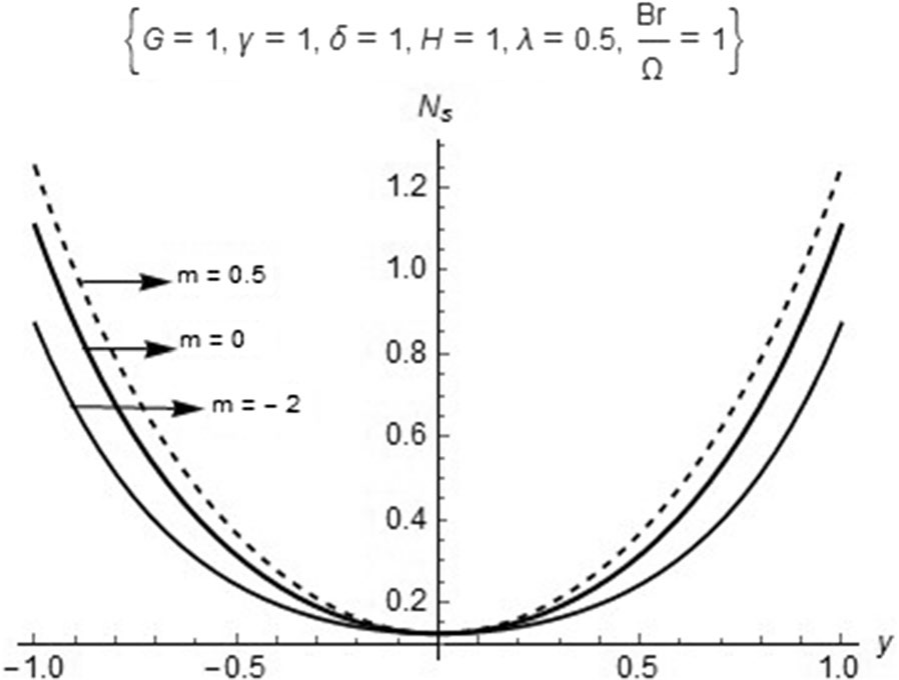
Comparison of entropy generation rates for different kinetics

The maximum temperature is observed in Fig. 4 as the numerical exponent (*m*) increases with the effect of the internal heat generation $(\beta )$, it is clearly seen that the heat generated internally contributes to an increase in the temperature of the fluid flow. The comparison of entropy generation rate for different chemical kinetics is shown in Fig. 5. It is observed that an increase in the numerical exponent (*m*) gives an increase in the entropy generation rate. Although, the entropy generation rate is at minimum which is above zero due to the effect of internal heat generation rates around the core region of the channel and rises to the maximum value of the plate surfaces.

**Fig. 6:**
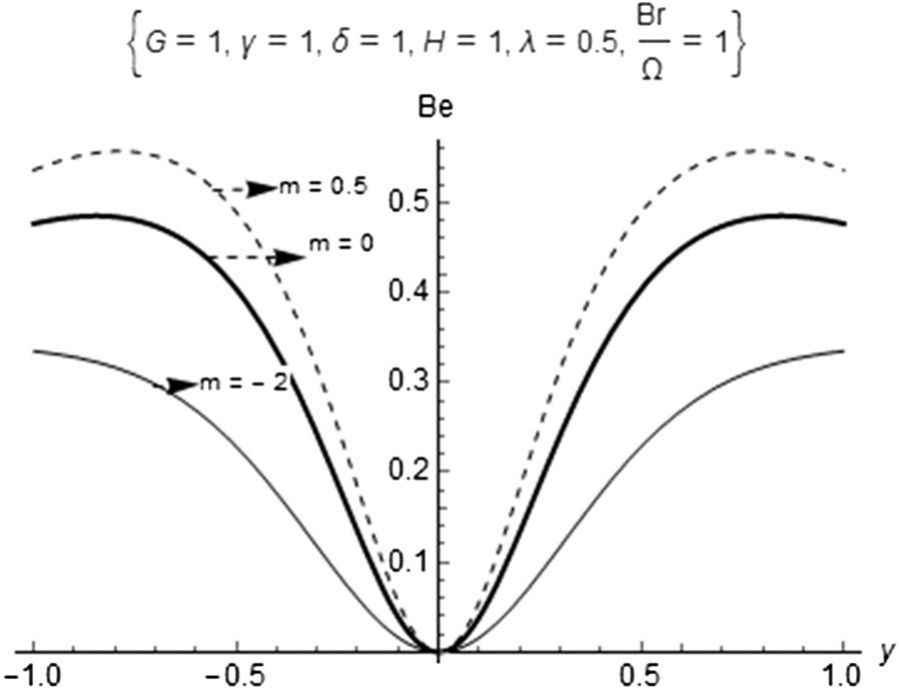
Bejan number for different chemical kinetics

**Fig. 7:**
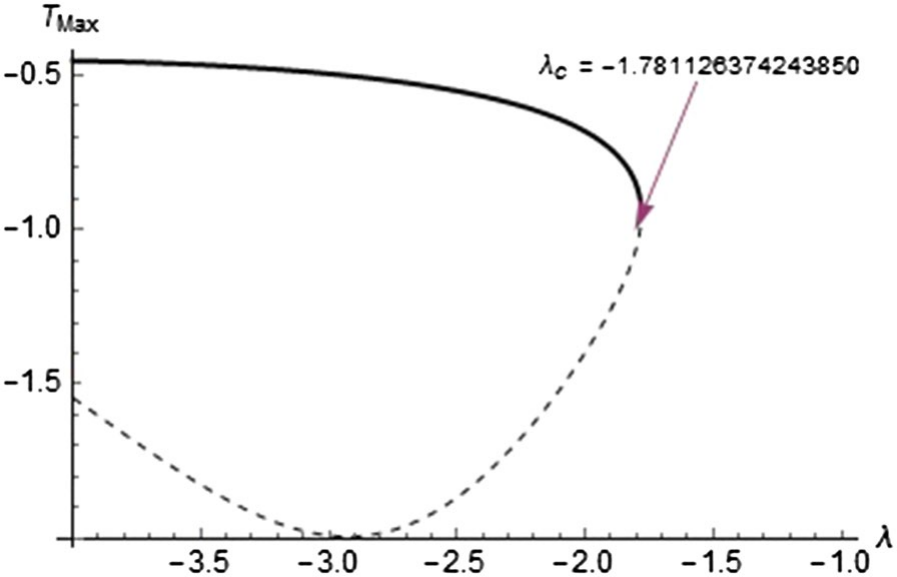
Solution branches for sensitized kinetics

**Fig. 8:**
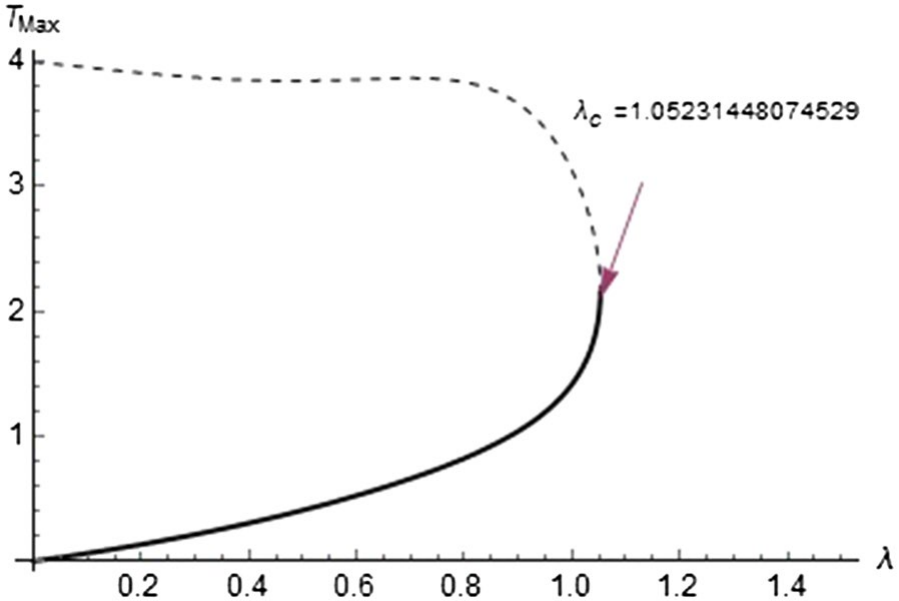
Solution branches for Arrhenius kinetics

Figure 6 also displays the comparison of the entropy generation rate for different chemical kinetics where heat transfer irreversibility dominates at both lower and upper plate surfaces and increase with increasing values of each numerical exponent (*m*) while fluid friction irreversibility dominates around the core region.

**Fig. 9:**
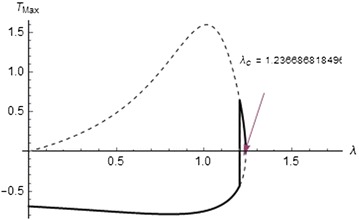
Solution branches for bimolecular kinetics

Figures 7, 8, and 9 show the solution branches for different chemical kinetics. These plots display the qualitative change in the flow system due to the effect of internal heat generation rates. The Frank-Kamenettski parameter $(\lambda )$ and each turning point $(\lambda _c)$ increase with respect to the numerical exponents from $-2$ to 0.5.

## Conclusion

The analysis of a reactive hydromagnetic internal heat generating Poiseuille fluid flow through a channel is carried out. The ADM was used to obtain the analytical solutions of the governing equations and the Pade approximation technique was used to determine the thermal criticality of a reactive hydromagnetic fluid flow through a channel for different chemical kinetics.

The results revealed that as the numerical exponent $m \in \left\{ -2, 0, 0.5\right\}$ increases the temperature also increases and that the effect of the heat source influenced the fluid flow by increasing the fluid temperature and that an increase in the magnetic field intensity increases the thermal criticality values. The entropy generation rate is observed to be at the minimum around the core region of the channel and rises to its maximum values at the plate surfaces and that an increase in the numerical exponents gives an increase in the entropy generation rate. It is found that among others, the thermal criticality conditions and with the right combination of thermophysical parameters controlling the system, the thermal runaway can be prevented. These will be of interest to lubrication companies in improving the efficiency and effectiveness of hydromagnetic materials used in engineering systems.
